# Assessment of the time course of chronic inflammation in the murine house dust mite model

**DOI:** 10.1186/1476-9255-10-S1-P1

**Published:** 2013-08-14

**Authors:** Stephen M Jordan, Robert Carrington, David Lanham, James Cartwright, Rachel A Armstrong, Kenneth G Meecham

**Affiliations:** 1 Department of Pharmacology, Huntingdon Life Sciences, Cambridgeshire, UK; 2 Department of Biomarkers, Bioanalysis and Clinical Sciences, Huntingdon Life Sciences, Cambridgeshire, UK; 3 Department of Pathology, Huntingdon Life Sciences, Cambridgeshire, UK

## 

House dust mite (HDM) allergens are associated with allergic disorders and the use of this clinically relevant allergen is increasing in animal models. We assessed the chronic inflammatory time course and the anti-inflammatory efficacy of a phosphodiesterase 4 inhibitor and a corticosteroid. BALB/c mice were challenged intranasally with HDM for 5 days/week for 5 weeks. Animals were sacrificed weekly 24 hours after final challenge and recruitment of inflammatory cells assessed in bronchoalveolar lavage fluid (BALF). Lung tissue was stained for the evaluation of a histopathological response. Roflumilast (10 mg/kg) and prednisolone (10 mg/kg) were administered orally twice daily from Week 3. Chronic HDM extract exposure resulted in significant recruitment of eosinophils, neutrophils, lymphocytes and macrophages as early as Week 1, peaking (1.13±032, 0.31±0.05, 0.66±0.10 and 0.33±0.04 x10^6^cells/animal respectively) between Weeks 3 and 5. Within the lymphocyte population the proportion of B cells increased from 4 to 46% over the 5 week exposure period. Mice developed perivascular, peribronchiolar and alveolar inflammation which increased in severity during the five week exposure period. The inflammation was accompanied by epithelial and mucus cell hypertrophy/hyperplasia in the bronchi and bronchioles which reached maximum severity during Weeks 3 to 5. Perivascular/peribronchiolar fibrosis peaked in Week 5. Therapeutic treatment with prednisolone and roflumilast significantly (P<0.001) inhibited BALF cell recruitment and reduced the severity of the airway remodelling suggesting this model, in our laboratory, has the potential to test novel compounds for the treatment of allergic disorders.

